# Implantation site design for large area diamond quantum device fabrication

**DOI:** 10.1038/s41598-023-40785-3

**Published:** 2023-08-18

**Authors:** Milan Vićentijević, Milko Jakšić, Tomislav Suligoj

**Affiliations:** 1 Ruđer Bošković Institute, 10000 Zagreb, Croatia; 2 Department of Electronics, Microelectronics, Computer and Intelligent Systems, Faculty of Electrical Engineering and Computing, University of Zagreb, 10000 Zagreb, Croatia

**Keywords:** Qubits, Characterization and analytical techniques, Design, synthesis and processing, Imaging techniques, Experimental particle physics

## Abstract

With the number of qubits increasing with each new quantum processor design, it is to be expected that the area of the future quantum devices will become larger. As diamond is one of the promising materials for solid state quantum devices fabricated by ion implantation, we developed a single board diamond detector/preamplifier implantation system to serve as a testbed for implantation sites of different areas and geometry. We determined that for simple circular openings in a detector electrode, the uniformity of detection of the impinging ions increases as the area of the sites decreases. By altering the implantation site design and introducing lateral electric field, we were able to increase the area of the implantation site by an order of magnitude, without decreasing the detection uniformity. Successful detection of 140 keV copper ions that penetrate on average under 100 nm was demonstrated, over the 800 µm^2^ area implantation site (large enough to accommodate over 2 × 10^5^ possible qubits), with 100% detection efficiency. The readout electronics of the implantation system were calibrated by a referent ^241^Am gamma source, achieving an equivalent noise charge value of 48 electrons, at room temperature, less than 1% of the energy of impinging ions.

## Introduction

Quantum centers in diamond have been widely investigated for both quantum optics^1^ and quantum computing applications^2–4^, due to their versatility^5,6^ and room temperature stability^7^. Among many different quantum centers in diamond, one of the most well-known is nitrogen vacancy (NV) center. NV center satisfies all criteria^8^ for qubit realization, which include: long decoherence time^9^, the ability to initialize the quantum system^10,11^ and possibility to create multiple coupled quantum centers^12^. To create a quantum device, arrays of closely packed quantum centers are required, in order to achieve strong coupling between individual centers. To achieve this in a solid-state substrate, we need to precisely place each implanted ion within the implantation site. This technique, where both the position and the number of implanted ions is controlled, is called deterministic implantation. One of the ways to achieve deterministic ion implantation is using focused ion beam implantation^13–15^ combined with ion detection (by detection of secondary electrons^16^, photons^17^ or free charge carriers in the substrate itself^18,19^). For deterministic implantation it’s crucial to obtain near 100% detection efficiency i.e., to be able to detect every single ion with high certainty. The most frequently used method is based on secondary electron detection, with efficiency up to 100% for implantation of heavy^20^ or high energy^21^ ions. Unfortunately, the number of emitted electrons from the sample surface becomes very low for lighter ions (such as N) with energy in the keV range^22^, lowering detection efficiency. Using an active substrate for detection of impinging ions proved to be a more successful approach^23^, with low energy P ions detected in Si by ion beam induced charge (IBIC) method with high efficiency^18^. So far, there were no publications about the similar results being achieved with diamond substrate. There are also methods based on pre-detection of ions, such as using Paul traps as source of single ions^24^ and detection of fly-by ions by image charge detection^25^. While the usage of Paul traps eliminates the difficulty of detecting low energy ions, it can’t provide the high implantation rate needed for efficient fabrication of devices with large number of quantum centers. On the other hand, detection of image charge is a promising method; however, it works efficiently only with packets of ions.

Unfortunately, the lateral and longitudinal straggling of the implanted ions increase with the ion energy, limiting the spatial precision of implantation. High spatial precision can only be achieved by implanting low energy ions that have low penetration range^26^, but also induce lower signal as they transverse the semiconductor material, compared to the more energetic ions, making them more difficult to detect by an active substrate. In the previous work we demonstrated detection of 140 keV copper ions in diamond^27^, showing that state-of-the-art commercial electronics and diamond crystal can be used for sub 100 nm ion implantation. In the presented work we focus on diamond detector design suitable for implantation over large areas. Large area, uniform implantation sites with high detection efficiency are a necessity for efficient and cost-effective fabrication of large quantum devices or multiple devices on a single substrate. So far the implantation sites (for silicon substrate) were of relatively small area (multiple sites of around 180 µm^2^ each^19^). Our aim was to propose the design for a large area (> 500 µm^2^) implantation site that can accommodate large number of quantum centers and have the capability to detect ions with sub 100 nm range across the whole surface.

## Results

**Figure 1:**
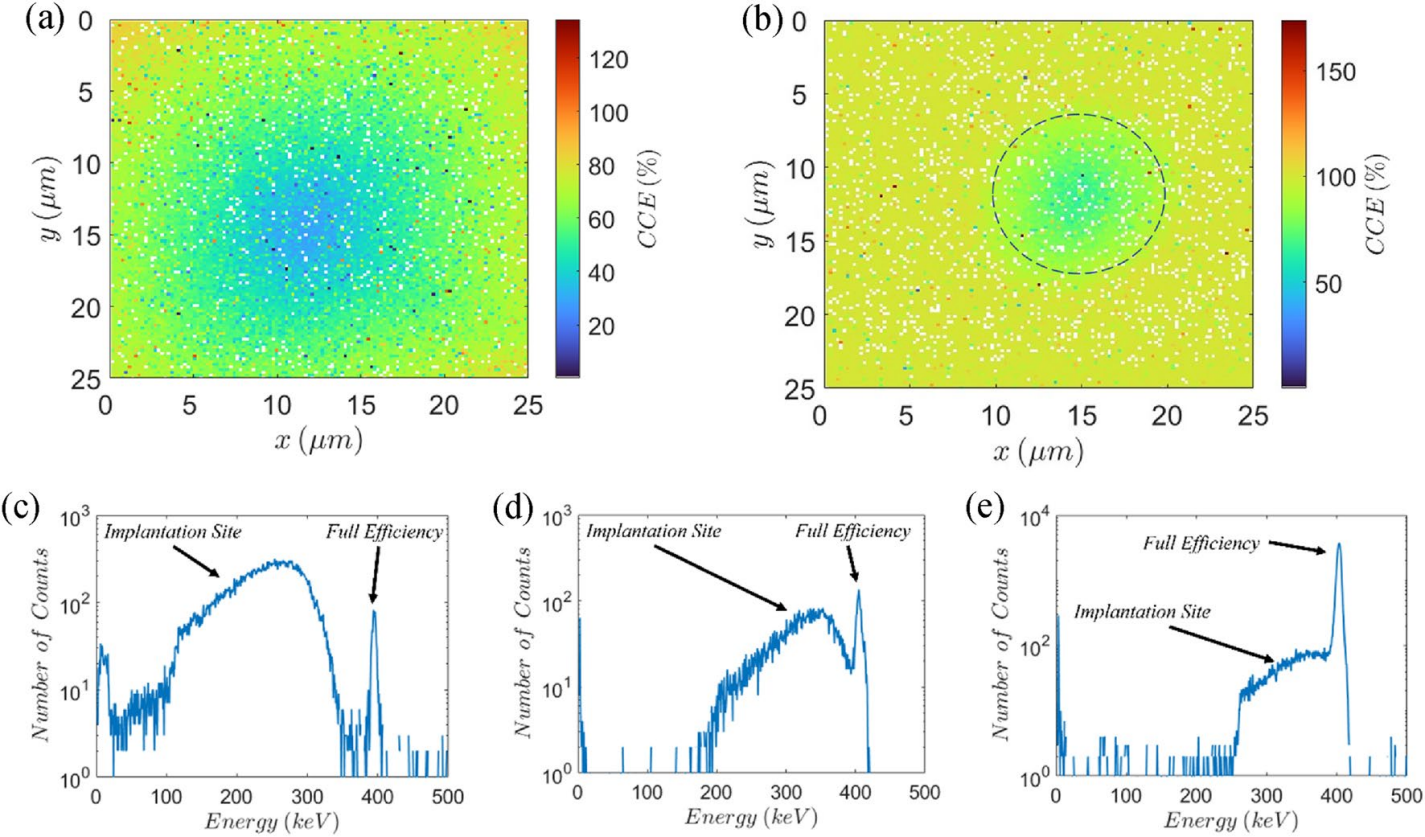
CCE distribution and histograms of different implantation sites. CCE map of (**a**) 50 µm and (**b**) 10 µm implantation site. Dashed line in (**b**) represents the edge of the hole in the sensing electrode. Histograms for (**c**) 50 µm (corresponding to CCE map in (**a**)), (**d**) 30 µm and (**e**) 10 µm (corresponding to CCE map in (**b**)) implantation site. The biasing voltage on the bottom electrode was set to $-150\,\mathrm{ V}$ in all cases. In (**c**–**e**) two distinct parts of spectrum were marked: one corresponding to signals originating from the implantation site ($\mathrm{CCE}<100\mathrm{\%}$); and the full efficiency peak, originating from area outside the implantation site (where $\mathrm{CCE}=100\mathrm{\%}$). Signals higher than full efficiency peak, corresponding to $\mathrm{CCE}>100\mathrm{\%}$ are pileup events. For (**a**) and (**b**) color white was hard coded for $\mathrm{CCE}=0\mathrm{\%}$.

To examine the uniformity of different implantation sites of a diamond detector we used 400 keV protons and 140 keV Cu^2+^ ions to record charge collection efficiency (CCE) distribution maps. The CCE is defined as the percentage of the total charge created in the substrate (by an impinging ion) that is integrated by the preamplifier. Since diamond is an indirect semiconductor, the energy needed for a single electron hole pair creation (13 eV) is higher than the bandgap energy (5.47 eV), due to phonon interactions. We assumed that the total charge created by an impinging ion is equal to the ratio of the energy of the ion and the energy needed for a single electron-hole pair (EHP) creation. This assumption is valid for light ions while it gives the lower limit of the achieved CCE for heavier ions that lose part of their energy through processes that do not result in EHP creation. For the initial characterization of the detector, a 400 keV proton beam was used. There are two main advantages in using 400 keV protons as a probing beam: 1. Low energy protons cause negligible damage to diamond crystal so they can be used for probing the CCE without performance degradation. 2. 400 keV protons pass through the electrode but stop in the diamond substrate so full energy peak can be recorded with high CCE. Three different implantation sites were characterized by 400 keV protons: circular openings in the diamond’s top electrode, with diameter of 50 µm, 30 µm and 10 µm, in further text referred to as 50 µm, 30 µm and 10 µm implantation site, respectively. In all the measurements with 400 keV proton beam the bottom electrode biasing voltage was set to $-150\,\mathrm{ V}$, since that voltage was sufficient for achieving 100% CCE. Figure 1a,b show CCE maps for 50 µm and 10 µm implantation site, respectively, while Fig. 1c–e show histograms for 50 µm, 30 µm and 10 µm sites, respectively.

For more accurate comparison of the histograms obtained for different implantation sites the biasing voltage as well as the total irradiation area (25 × 25 µm^2^) was the same in all three cases. This enables 100% CCE (full efficiency) peak to be recorded alongside the signals for 30 µm and 10 µm implantation sites, since their area is smaller compared to the irradiation area. Even though the irradiation area is smaller than the area of the largest implantation site, the 100% CCE peak is still visible in all three histograms. This is due to the beam halo, which spans outside the irradiation area. In general, beam halo is undesirable and different techniques are used to suppress its influence. However, in the case of the 50 µm implantation site it is useful since the full efficiency peak in histogram in Fig. 1a is a consequence of beam halo. It is important to state that the existence of beam halo has minor influence on the overall spectrum in all three cases, since the number of events corresponding to beam halo is small in comparison to the total number of events: $<2\%$ of the total number of events in case of the 50 µm implantation site and $<1\%$ of the events corresponding to the full efficiency peak for the 10 µm one.

**Figure 2:**
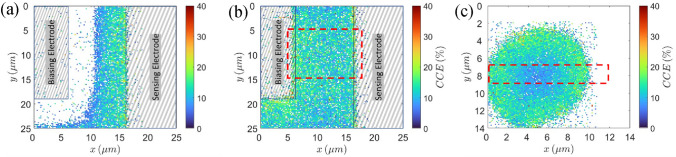
CCE distribution maps obtained by 140 keV copper ions. (**a**) Interstrip region for biasing electrode at 0 V; (**b**) interstrip region for biasing electrode at $-180\,\mathrm{ V}$; (**c**) 10 µm implantation site. Color white was hard coded for $\mathrm{CCE}=0\mathrm{\%}$.

In the second implantation campaign we compared CCE distribution of an 80 × 10 µm^2^ implantation site, between two strip electrodes (interstrip region), to a CCE distribution of a 10 μm circular one (hole). We chose that specific hole since it has the best CCE uniformity of all the circular implantation sites, and its diameter is the same as the distance between the strips of the interstrip region. The ion beam used for this campaign was 140 keV Cu^2+^. It is the least penetrating ion beam our accelerator and focusing system can provide, with penetration depth in diamond (59 ± 16 nm as calculated by SRIM^28^) almost identical to the penetration depth of 50 keV nitrogen ions (62 ± 15 nm) the ion beam needed for creation of precisely placed NV centers in diamond. For both the hole and interstrip implantation the voltage on the bottom electrode was set to $-250\,\mathrm{ V}$ (the maximum voltage the biasing board can sustain), which creates very high electric field across the crystal (> 6 V/µm), since it is only 40 μm thick. The voltage on the biasing electrode (biasing strip of the interstrip implantation site) was between 0 and $-180\,\mathrm{ V}$. Figure 2a,b show CCE distribution maps of two extreme cases of the lateral electric field across the interstrip region, while Fig. 2c shows CCE distribution map for 10 µm hole. In this case beam halo has no influence on the CCE maps since it spans outside the implantation sites where the ions can’t penetrate the electrode therefore, no beam halo events can be detected.

## Discussion

Comparison of the histograms obtained for different implantation sites by 400 keV proton beam (Fig. 1c–e) shows that as the area of the sites decreases their signal becomes higher and more uniform. In Fig. 1c the signal from the 50 µm implantation site is fully separated from the full efficiency peak and very wide (> 250 keV). Signal from the 30 µm site (Fig. 1d) is narrower (~ 200 keV) and overlaps with the full efficiency peak, but the two peaks can still be resolved. However, in case of 10 µm hole, the implantation site signal cannot be resolved from the full efficiency peak but is only present as a 150 keV wide tail to the main peak. Therefore, the smallest implantation site has not only the highest probability of ion detection but the best CCE uniformity, as well. Unfortunately, the cost of that is the smallest active area.

**Figure 3:**
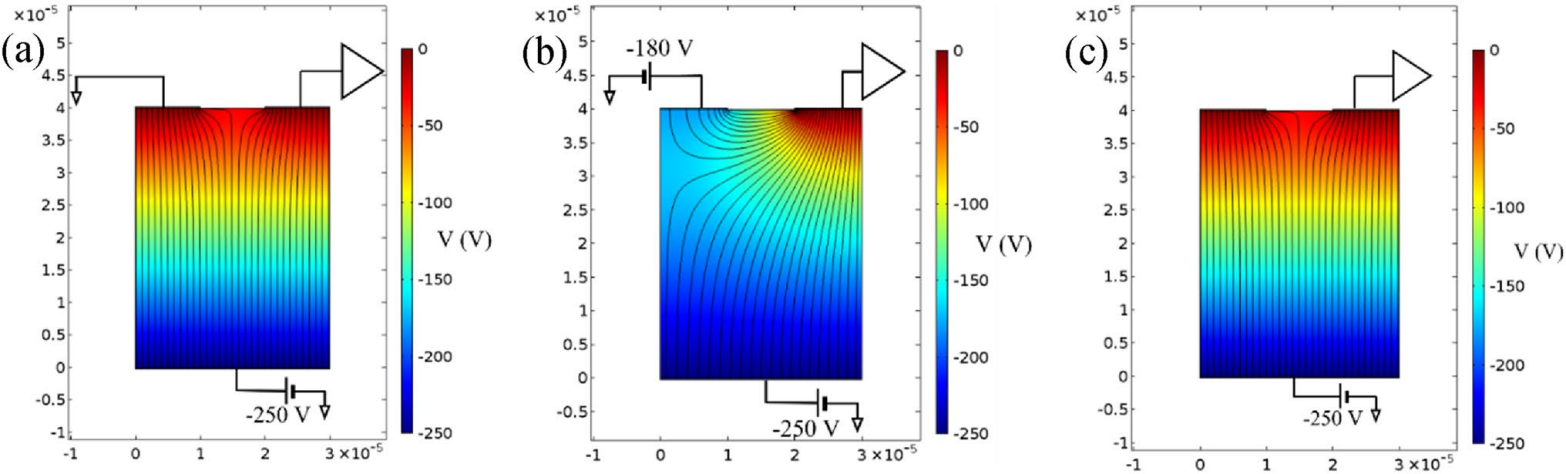
Electric potential (magnitude indicated by color) and field (magnitude proportional to line density) simulation. (**a**) Interstrip region for biasing electrode at 0 V; (**b**) Interstrip region for biasing electrode at $-180\,\mathrm{ V}$; (**c**) 10 µm hole. x and y axis are in meters.

Unlike the probing with 400 keV protons, the implantation of 140 keV copper ions to the interstrip implantation site and the 10 µm one was not done with same beam implantation area, although the bias from the bottom electrode was the same for both sites ($-250\,\mathrm{ V}$). Since 140 keV copper ions can’t penetrate the electrode, implantation through the electrode material itself was not an option, so we decided to reduce the implantation area for 10 µm site to get a more detailed CCE map. For biasing electrode at 0 V, (Fig. 2a) there is a large dead zone (zone where CCE is zero) spanning from the middle of the implantation site to the biasing electrode. There is no dead zone when the biasing electrode voltage is set to $-180\,\mathrm{ V}$ (Fig. 2b). To try to explain the behavior of the dead zone we simulated electric potential and field distribution in diamond using COMSOL^29^. Figure 3a–c show the simulations for interstrip region without the lateral electric field, with the lateral electric field, and for the 10 µm hole, respectively. For simulation purposes we assumed that the sensing electrode is at ground potential. Even though the sensing electrode potential is undefined (directly connected to the preamplifier input), it usually is around 0 V^27^. Figure 3a shows divergence in the electric field lines around the middle point between the strips. Therefore, the free charge carriers that are created close to the sensing electrode would drift towards that electrode inducing a measurable signal, while the free charge carriers created closer to the bias electrode would drift towards it (away from the sensing electrode) inducing negligible signal in the sensing electrode. The electric field and potential distribution in case of a 10 µm implantation site (Fig. 3c) is the same, but without the dead zone, because the sensing electrode in that case is all around the hole, so the free charge carriers always drift towards the sensing electrode, inducing a measurable signal. In the case with lateral electric field (Fig. 3b), the electric field under the diamond surface is more uniform and directed towards the sensing electrode, a favorable setup for detection of small range ions.

CCE value of around 20% is recorded along the edge of the sensing (biasing and sensing) electrode without (with) lateral electric field, with a slight drop towards the center in case when lateral electric field is present. Even though there is no significant difference in the maximum value of CCE, the CCE distribution is much more uniform across the implantation site with the lateral field present. The CCE distribution within the 10 μm hole, shown in Fig. 2c, shows CCE values of close to 20% in areas with distance of 1 µm or less to the hole edges. CCE then drops below 10% at the center of the implantation site. It should be noted that detection efficiency in case of implantation site with the lateral electric field and 10 µm hole is 100%, that is, every single ion is detected, which can be seen from CCE maps, that show no dead zones.

**Figure 4:**
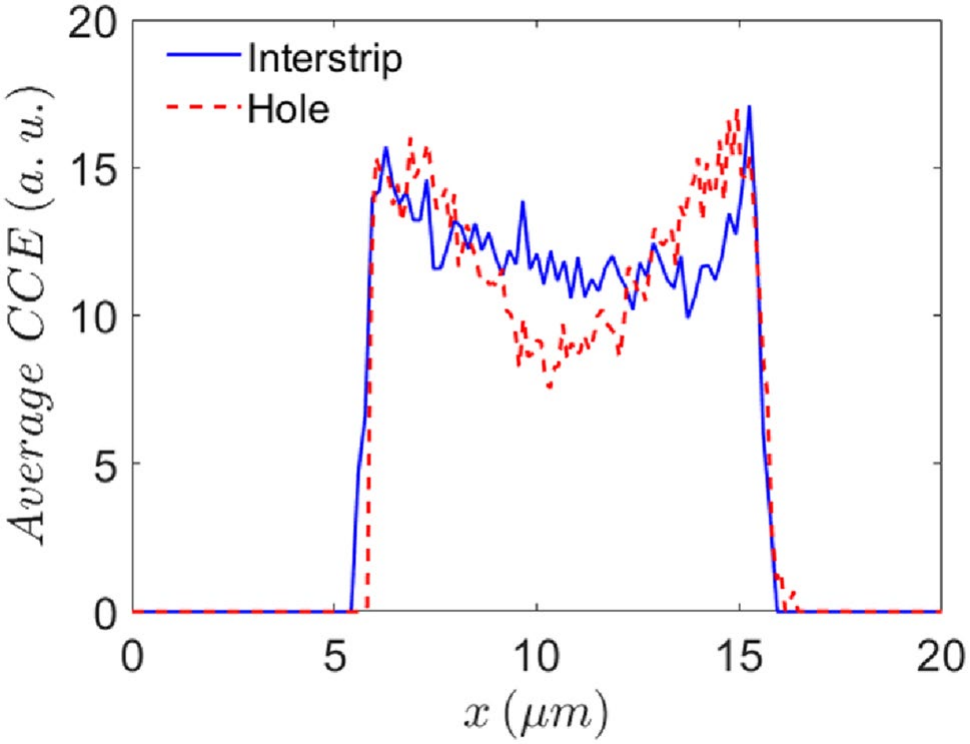
Pointwise average CCE for interstrip implantation site and 10 µm hole regions marked by a red dashed rectangle in Fig. 2b and c, respectively. Padding (leading and trailing zeros) was added to the datasets so they can be overlayed correctly.

We compared the magnitude of the CCE across the interstrip implantation site and the 10 μm one (Fig. 4). The graph shows the pointwise average CCE in the region indicated by the red dashed rectangle in Fig. 2b and c, respectively. The region of interest was 10 μm × 12 μm for the interstrip site, and 2 μm × 12 μm for the 10 µm hole and was positioned across the center of the hole. To display the overlay of the two regions of interest correctly (one over the other) padding was added to the corresponding datasets in form of leading and trailing zeros. This compensates for the difference in the number of datapoints in the two datasets without altering the data itself. Even though interstrip implantation site has area an order of magnitude higher than the 10 µm site it shows better overall CCE uniformity, without such prominent CCE drop in the middle. Unfortunately, the CCE value in case of 140 keV copper ions is very low, on average under 20% in both interstrip and 10 µm implantation site. This is somewhat expected because of the pulse height defect which is significant for heavy ions, such as copper. The energy lost to nuclear collisions is more significant for low energy heavy ions. Moreover, heavy ions create denser free electron hole pairs cloud causing recombination of some of the free charge carriers quickly after their creation. Both effects lead to lower CCE compared to the lighter ions^30^. Another possible explanation for low CCE value is that unterminated diamond surface acts as electron sink^31,32^ because of the negative electron affinity, decreasing the number of free charge carriers created by the shallow copper ions. Supporting that claim is the fact that there is no significant difference in the CCE value between the two implantation sites even though their geometry and consequently electric field distribution is quite different. Even though the magnitude of the CCE is similar for both the 10 µm and the interstrip implantation site, the uniformity of the CCE differs. Towards the middle of the hole the CCE decreases faster than it does for the interstrip region. Therefore, if an ion is implanted in the center of the hole, its corresponding signal would be lower compared to the signal corresponding to an ion implanted close to the edge of the hole or to an ion implanted in the interstrip region.

Successful detection of low energy, shallow ions across the whole implantation site is an important step in the deterministic implantation process. It strongly depends on low noise readout electronics and electrode layout that enhances CCE. The overall noise of the implantation detector was less than 1% of the energy of copper ions used in the measurements, while the noise of the readout system was only 48 electrons. We've shown that small diameter openings in the sensing electrode can serve as simple and effective implantation sites. However, the decrease in CCE value close to the center of the hole would significantly lower the chances of ion detection. Therefore, we propose interstrip implantation site configuration with both vertical and lateral components of the electric field. In that way a large implantation site with higher CCE uniformity can be created. This is very important when implanting large arrays of low energy ions. For example, the proposed 800 µm^2^ implantation site can accommodate more than 2 × 10^5^ points of implantation of 50 keV N ions that can create NV centers upon activation. That means up to 2 × 10^5^ possible qubits. In this estimate we assumed that the stationary position of each implanted ion, within the diamond crystal, would be within a cylinder whose base is a circle with center at the point of implantation and diameter of 62 nm, equal to the longitudinal range of the ions (a conservative estimate since the lateral straggling of N ions is smaller than their range.). The same number was used for the distance between two implantation points in x and y direction (plane defined by the crystal surface) that ensures no possible overlapping of quantum center positions.

The CCE uniformity is also important for using the proposed geometry with other ion species, where noise is the limiting factor. As already mentioned, for 140 keV copper ions the system demonstrates 100% detection efficiency therefore, the same efficiency is to be expected for other ion species with penetration depth in diamond higher or equal to 140 keV Cu ions. Examples of such ion species that can be used for quantum center creation in diamond are: 170 keV Ge, 90 keV Si, 65 keV Mg and 50 keV N, all having penetration depth of around 60 nm in diamond. The detection limit of the system can be written as:

1 $$\frac{{E}_{Ion}}{{E}_{EHP}}\cdot CCE>3 {e}_{in}$$

where ${E}_{Ion}$ is the energy of the impinging ion, ${E}_{EHP}$ is the energy needed to create one EHP in diamond, $CCE$ is charge collection efficiency and ${e}_{in}$ is equivalent noise charge at the input of the preamplifier in number of electrons (noise figure of the detector/preamplifier system). For separation of signal from noise in data histogram, there should be $6\sigma$ separation distance between the average signal amplitude and the average noise level. In that way the overlap of signal and noise happens in only 0.15% of the recorded events. The relation between full width at half maximum (FWHM) of the signal (the value we used as the noise figure) and the standard deviation ($FWHM\approx 2.355 \sigma$) means that $6 \sigma \approx 2.55 FWHM$. For a more conservative estimate of the lowest energy, we decided to set the threshold at $3 FWHM$. Equation (1) can also be interpreted in terms of signal-to-noise ratio (SNR) where $SNR>3$ for high fidelity detection. Since the proposed system has 100% detection efficiency and > 10% CCE for high lateral electric field implantation site, for ion species that penetrate around 60 nm or more, the minimum detectable energy is 18.7 keV. This means that the described detector/preamplifier system should have no problem detecting 170 keV Ge, 90 keV Si, 65 keV Mg or 50 keV N ions. For ions of lower energy and penetration depth, CCE is expected to be lower which would increase the minimum detectable energy. Because CCE does not decrease linearly with the penetration depth it is very difficult to predict the absolute limit of the system i.e., the minimum energy of the impinging ions the system can detect with 100% detection efficiency.

The proposed system is relatively cheap, easily transported and fully compatible with other semiconductor materials interesting for quantum device fabrication (such as Si or SiC). Moreover, by implementing the proposed implantation site geometry into interdigitated electrode configuration, the implantation area can be extended to cover nearly the whole surface of the sample, enabling better diamond utilization. The main advantage of the proposed technique is that the implantation process, even for 2 × 10^5^ implants, can be completed in a matter of hours and requires no special sample preparation or cooling but can be done at room temperature. This is both more cost effective and energy efficient compared to quantum center fabrication based of large equipment such as ion traps which have a limited ion capacity and cost several orders of magnitude more. Activation of implanted ions to functioning quantum centers with high efficiency remains an important task in large scale quantum device fabrication. However, advances in detection of low energy ions could contribute to finding the optimal parameters (such as optimal number of implanted ions per implantation site) for more efficient ion activation.

## Methods

**Figure 5:**
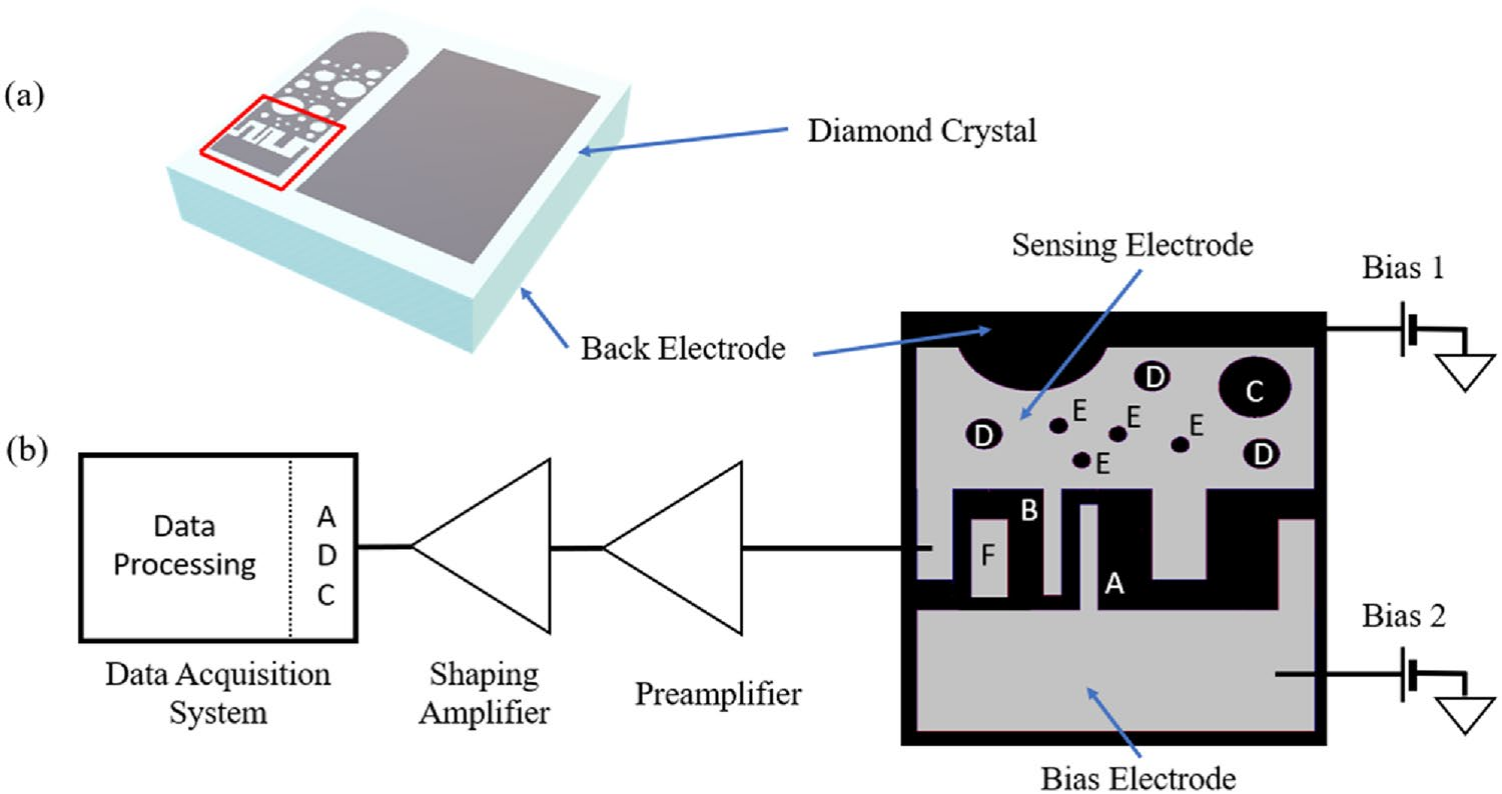
Diamond detector layout. (**a**) Schematic diagram of the diamond sample with two electrodes used in the measurements marked by a red rectangle. (**b**) Layout of the two electrodes used in the measurements together with the readout electronics connection scheme.

The results presented in this paper were obtained by using focused ion beams and IBIC technique^33,34^ to detect the signal induced in the substrate by each impinging ion. The active substrate was high purity (impurity concentration < 5 ppb), high resistivity (> 10^11^ Ω m), single crystal, electronic grade diamond, synthesized by chemical vapor deposition, produced by Element Six Ltd. The ions used were 400 keV protons and 140 keV Cu^2+^ ions with average range in diamond of 2100 ± 52 nm and 59 ± 16 nm, respectively, as calculated by SRIM^18^. The diamond sample, shown in Fig. 5a, is a 4 × 4 × 0.04 mm^3^ crystal with a single electrode on the bottom side and four electrodes on the top side, mounted on an AlN printed circuit board (PCB). The AlN PCB has high thermal conductivity (321 W/(m K)) and heat resistance (> 1000 °C) which makes it suitable for sample cooling or high temperature thermal annealing. However, for annealing of the sample all elements of the system should be heat resistant. That includes adhesive used for attaching the sample to the PCB and diamond electrodes (which can be made of tungsten). The readout electronics are not heat resistant so that part of the system should be detached before annealing, which can be done by incorporating trenches between the detector part and preamplifier part of the PCB. After the implantation, the detector part could be easily broken off along the line of trenches (AlN is very brittle) without the risk of damaging the sample. The electrodes are made of 200 nm thick aluminum and were fabricated in Diamond Sensors Laboratory, CEA-LIST Institute, Paris. The largest top electrode is 3.8 × 1.3 mm^2^ and is used for measurements in standard, planar mode. The other three top electrodes were designed with several implantation sites of different dimensions and electric field distribution. In the research presented in this paper only two top electrodes were used (indicated by a red rectangle on Fig. 3a), together with the bottom electrode. The implantation sites are realized as circular openings—holes, in the sensing electrode. There are total of 8 holes: one with 50 μm diameter (marked with C), three with 30 μm diameter (marked with D) and four with 10 μm diameter (marked with E). For all holes the biasing voltage is connected to the bottom electrode creating quasi vertical electric field. Part of the sensing electrode is also a 10 × 100 μm^2^ strip (marked with B). The sensing electrode is directly bonded to the XGLab CUBE PRE_031 low noise preamplifier, mounted on the same AlN PCB as the diamond itself, and connected to the XGL-CBB-1CH biasing board. The other top electrode used in the measurements is connected to the biasing voltage and thus called bias electrode. It also has a 10 × 100 μm^2^ strip (marked with A), same as the sensing electrode. The two strips are 10 μm apart, and together make a 10 × 80 μm^2^ implantation site. For this site biasing voltage is connected to the bottom electrode, same as for holes but there is also a possibility of applying additional biasing to A strip creating a lateral electric field component across the implantation site. There is also a floating rectangular electrode (marked with F), which was not used. Detailed illustration of the two top electrodes of interest, as well as their connection to the readout system, is given in Fig. 5b. Voltage on the bottom electrode can have values between 0 and $-250\,\mathrm{ V}$, while biasing electrode voltage can be set to any value between 0 V and the voltage on the bottom electrode. The signal from the preamplifier is processed by ORTEC 570 shaping amplifier, before digitization in Canberra 8075 ADC. Digitized data is then further processed and recorded, in real time, using a custom-made data acquisition system^35^.

**Figure 6:**
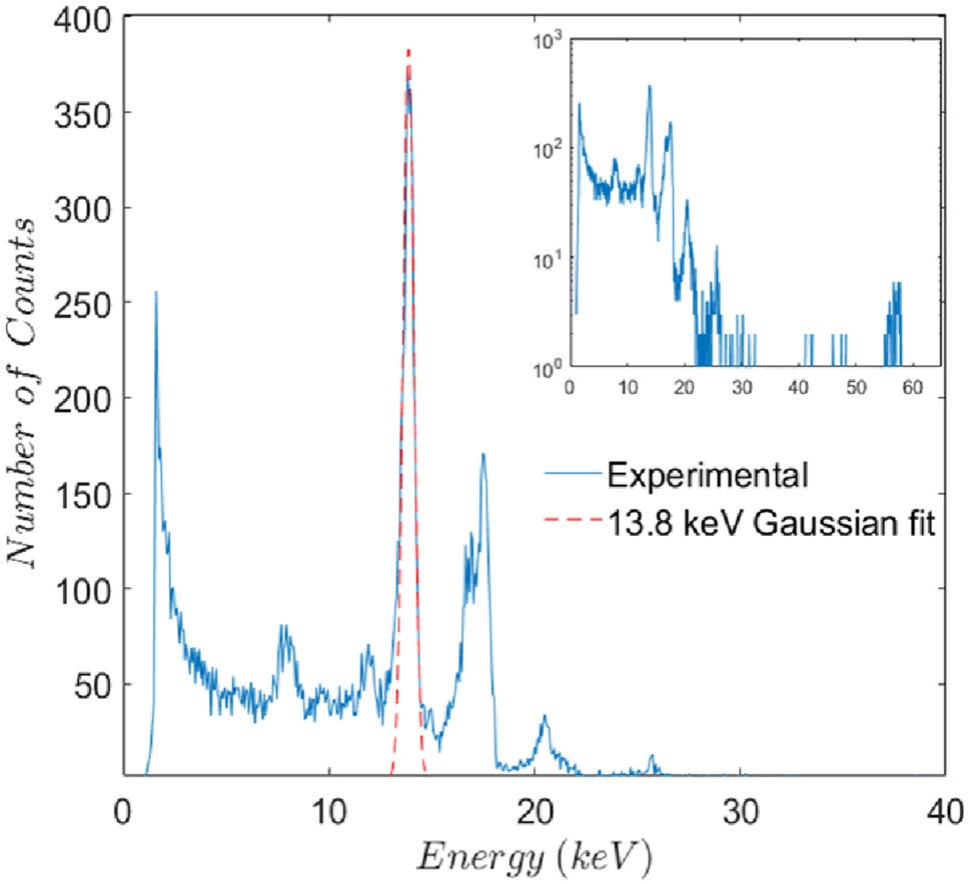
Histogram obtained from a referent ^241^Am gamma source by calibration diamond detector. Main figure shows histogram for energies between 0 and 40 keV, in linear scale. Full histogram in semilogarithmic scale shown as snippet. Gaussian fit for a 13.8 keV peak is shown by red, dashed curve.

For the energy calibration of the readout system, we used a referent ^241^Am gamma source. It emits photons of the following energies: 13.8 keV, 17.7 keV, 20.7 keV, 26.3 keV and 59.5 keV, with the last one being the dominant emission^36^. Due to the complex electrode layout of the implantation diamond and its small thickness, gamma detection wouldn’t result in distinct peaks. Therefore, we used another detector of the same quality, a 2 × 2 × 0.5 mm^3^ single crystal diamond, with planar electrode configuration (1 × 1 mm^2^ top and 2 × 2 mm^2^ bottom electrode). The calibration diamond was mounted on AlN PCB of the same kind as the implantation diamond and bonded to XGLab CUBE PRE_031 preamplifier. To ensure the same performance of calibration and implantation systems both the PCB and the preamplifier used for calibration purposes were from the same production lot as those used for implantation. Figure 6 shows histogram obtained by the calibration diamond for energies between 0 and 40 keV, with the full spectrum shown as snippet in semilogarithmic scale. Even though emission at 59.5 keV is dominant, that peak is two orders of magnitude smaller than the 13.8 keV and 17.7 keV peaks due to the poor detection efficiency of diamond at higher photon energies. The noise figure of the system with the calibration detector is 0.63 keV, calculated as full width at half maximum of the Gaussian fit for the 13.8 keV peak, and represents the energy resolution of the system. Energy resolution as noise figure is useful when comparing different detector/preamplifier systems of the same detection material, but of little use when comparing systems with detectors made of different semiconductors. To obtain a noise figure of the readout electronics, independent of the detector material we need to divide the 0.63 keV with the energy needed to create one electron hole pair in diamond (13 eV), thus we get the equivalent noise charge of 48 electrons. To calculate the energy resolution of the system with detector made of some other semiconductor material we just need to multiply the equivalent noise charge with the energy needed to create one electron hole pair in the material of interest. In that way we get the energy resolution of the readout electronics to be 0.18 keV and 0.38 keV if used with silicon and SiC detector, respectively. This, however, is only valid for small capacitance detectors ($\mathrm{C}<0.5 \; \mathrm{ pF}$) since the preamplifier is optimized for that capacitance.

## Data Availability

All data generated or analysed during this study are available from the corresponding author on reasonable request.
